# Using quantitative systems pharmacology modeling to optimize combination therapy of anti-PD-L1 checkpoint inhibitor and T cell engager

**DOI:** 10.3389/fphar.2023.1163432

**Published:** 2023-06-20

**Authors:** Samira Anbari, Hanwen Wang, Yu Zhang, Jun Wang, Minu Pilvankar, Masoud Nickaeen, Steven Hansel, Aleksander S. Popel

**Affiliations:** ^1^ Department of Biomedical Engineering, Johns Hopkins University School of Medicine, Baltimore, MD, United States; ^2^ Biotherapeutics Discovery Research, Boehringer Ingelheim Pharmaceuticals Inc., Ridgefield, CT, United States; ^3^ Department of Oncology, Sidney Kimmel Cancer Center, Johns Hopkins University School of Medicine, Baltimore, MD, United States

**Keywords:** immune-oncology, quantitative systems pharmacology (QSP), immune checkpoint inhibitor, bispecific T cell engager, virtual clinical trial, dose optimization

## Abstract

Although immune checkpoint blockade therapies have shown evidence of clinical effectiveness in many types of cancer, the outcome of clinical trials shows that very few patients with colorectal cancer benefit from treatments with checkpoint inhibitors. Bispecific T cell engagers (TCEs) are gaining popularity because they can improve patients’ immunological responses by promoting T cell activation. The possibility of combining TCEs with checkpoint inhibitors to increase tumor response and patient survival has been highlighted by preclinical and clinical outcomes. However, identifying predictive biomarkers and optimal dose regimens for individual patients to benefit from combination therapy remains one of the main challenges. In this article, we describe a modular quantitative systems pharmacology (QSP) platform for immuno-oncology that includes specific processes of immune-cancer cell interactions and was created based on published data on colorectal cancer. We generated a virtual patient cohort with the model to conduct *in silico* virtual clinical trials for combination therapy of a PD-L1 checkpoint inhibitor (atezolizumab) and a bispecific T cell engager (cibisatamab). Using the model calibrated against the clinical trials, we conducted several virtual clinical trials to compare various doses and schedules of administration for two drugs with the goal of therapy optimization. Moreover, we quantified the score of drug synergy for these two drugs to further study the role of the combination therapy.

## 1 Introduction

Colorectal cancer (CRC) is the third most frequent cause of cancer-related death worldwide (Bray et al., 2018). Surgery, chemotherapy, and radiotherapy—also used in combination—have historically been the standard treatments for colorectal cancer. Unfortunately, these treatments have a lot of adverse consequences since they are non-specific and cytotoxic to all cells, including healthy cells (Johdi and Sukor, 2020). In recent years, cancer immunotherapy as a more effective alternative approach has changed the area of cancer treatments (Morrissey et al., 2016; Golshani and Zhang, 2018).

Immune checkpoint blockade therapies, including anti-PD-L1 and anti-PD-1, have raised a lot of attention and have shown a significant increase in the survival rate of patients with multiple solid tumor types (Alsaab et al., 2017; Popovic, Jaffee, and Zaidi, 2018; Sharma and Allison, 2020; Sharma et al., 2021). Nonetheless, the results of clinical trials show that only a small number of patients with metastatic CRC (mCRC) benefit from checkpoint inhibitors (Hegde and Chen, 2020). For example, the IMblaze370 study failed to improve overall response in the PD-L1 inhibitor atezolizumab monotherapy or even in combination therapy with the MEK inhibitor cobimetinib when compared with regorafenib in previously treated mCRC patients (Eng et al., 2019). It is essential, however, to keep looking into the role of checkpoint inhibitors, particularly in combination with other immunotherapy methods for the treatment of colorectal cancer.

The T cell bispecific antibody, cibisatamab (CEA-TCB), is a novel immunotherapy agent that guides T cells to tumor cells that express the carcinoembryonic antigen (CEA) glycoprotein at the cell surface regardless of their T cell receptor specificity (Bacac et al., 2016; Lehmann et al., 2016; Gonzalez et al., 2019). Numerous colorectal tumors exhibit an overexpression of CEA on their cell surfaces, making cibisatamab a prospective candidate for the treatment of colorectal cancer. Cibisatamab (RO6958688; RG7802) has been used in monotherapy and in combination with atezolizumab (anti-PD-L1) in clinical trials (NCT02324257, NCT02650713). The results from these trials have shown promising outcomes for the treatment of CRC with bispecific antibodies in solid tumors (Tabernero et al., 2017).

Although the combination therapy of bispecific antibody with PD-L1 inhibitors in solid tumors has shown significant promise, there may be drawbacks down the road, including the inability to pinpoint the cause of side effects, drug-drug interactions, cumulative side effects, and greater costs. As a result, optimization of dose and sequence for these combination therapies can be beneficial to reduce the potential risk of combination therapies and enhance the advantages.

Moreover, identifying the combination therapies with synergistic effects, which enable dose reduction of individual drugs and increase their efficacy, is desirable in clinical studies specially for bispecific antibodies that show toxic behavior at higher doses. Several synergy quantification methods have been proposed to assess drug combination performance (Chou, 2010; Meyer et al., 2020). The majority of synergy metric approaches are based on either Loewe Additivity (LA) principle (Loewe, 1953) or Bliss Independence (BI) method (BLISS, 1939; Berenbaum, 1978; Greco et al., 1995). More recently, a synergy framework called multi-dimensional synergy of combinations (MuSyC) has been introduced (Meyer et al., 2019), which was used in this study to quantify the synergy of combination therapy with atezolizumab and cibisatamab. This method’s key benefit is its ability to distinguish between synergetic potency and synergistic efficacy.

In this study, we have extended our previously developed QSP model of T cell engager (TCE) and anti-PD-L1 antibody in CRC (Ma et al., 2020a; Ma et al., 2020b) by incorporating the dynamics of helper T cells (Th) and myeloid-derived suppressor cells (MDSCs) from our study of triple-negative breast cancer (TNBC) (Wang et al., 2021) and modified the binding dynamics of TCE to fit the *in vitro* data of cibisatamab (Vyver et al., 2021). Using this model, we aim to optimize the dose and sequence of cibisatamab and atezolizumab and investigate their synergistic behavior in combination therapy.

## 2 Materials and methods

### 2.1 Model structure

**FIGURE 1:**
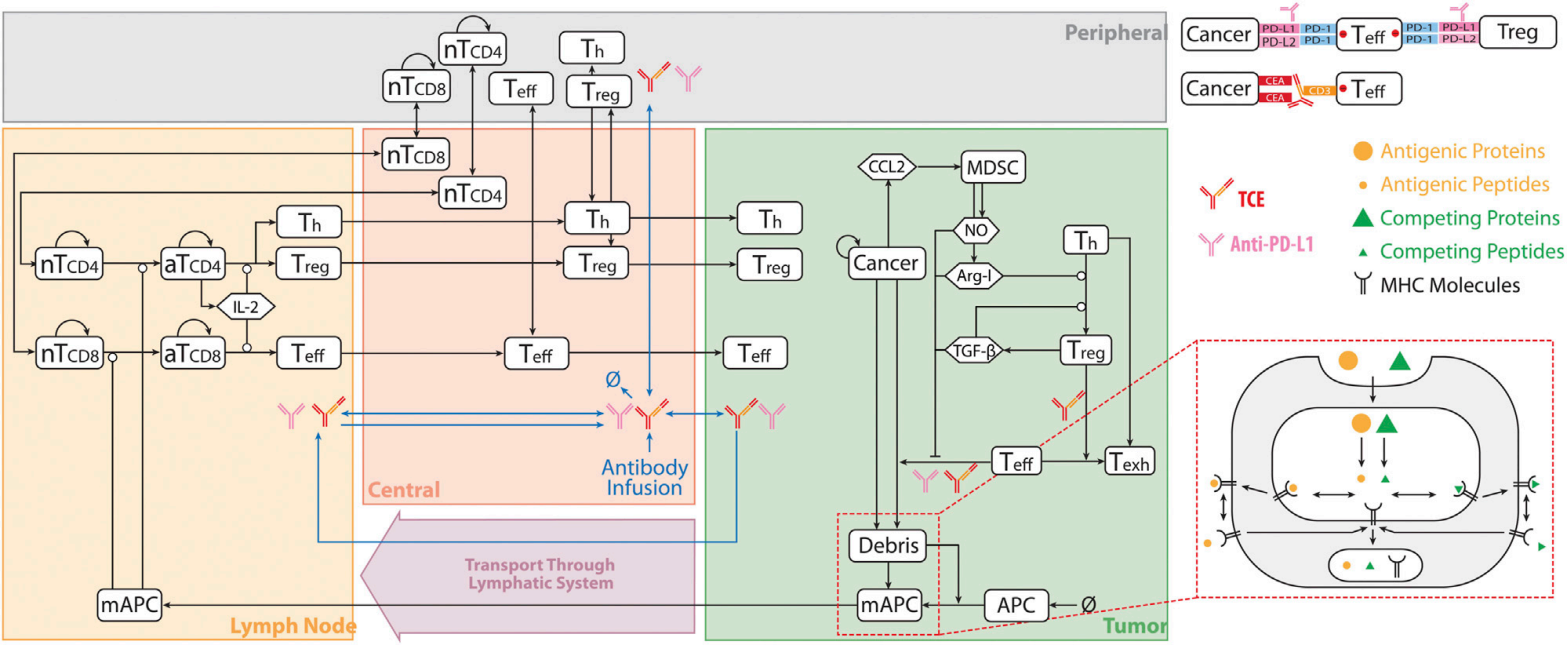
QSP Model Diagram. The model is divided into of four compartments: central, peripheral, tumor, and tumor-draining lymph node, which describe cycles of immune activation in lymph nodes, T cell trafficking to the tumor, killing of cancer cells, immune evasion, and antigen release and lymphatic transport. nT, naïve T cell; aT, activated T cell; NO, nitric oxide; Arg-I, arginase I; Treg, regulatory T cell; Teff, effector T cell; Th, helper T cell; Texh, exhausted T cell; MDSC, myeloid derived suppresser cells; mAPC, mature antigen presenting cell. Cytokine degradation and cellular clearance were omitted in the diagram. Modified from (Ma et al., 2020b; Wang et al., 2021).

The current QSP model is modified from our previously published QSP platforms (Ma et al., 2020a; Wang et al., 2021) built using SimBiology toolbox in MATLAB (MathWorks, Natick, MA). The model is composed of four compartments, which includes central, peripheral, tumor and tumor-draining lymph node compartments. The model consists of connected modules that describe the dynamics of molecular and cellular interactions associated with different species shown in Figure 1. In summary, the model simulates the dynamics of naïve CD4^+^ and CD8^+^ T cells, taking into account their trafficking between the central, peripheral, and lymph node compartments, as well as their proliferation in the peripheral and lymph node compartments. A small number of cancer cells are initially incorporated into the tumor compartment, and their dynamics is modeled using a logistic growth approach. Cancer cell death, or apoptosis, is modeled as a first-order reaction, which results in the release of tumor-associated neo-antigens and self-antigens into the tumor compartment.

The model considers the uptake of tumor-derived neo-antigens and self-antigens by antigen-presenting cells (APCs), their subsequent maturation, and their migration to the tumor-draining lymph node compartment. The detailed mechanisms of antigen processing and presentation, including the cleavage of proteins into peptides, binding of peptides to MHC molecules, and transport to the cell surface, are all incorporated into the model. The activation of naïve T cells is dependent on the extent of T cell receptor ligation by peptide-MHC on APCs, and is implemented as a Hill function. Following activation, Tregs, cytotoxic T cells, and helper T cells infiltrate into the tumor. Tumor-infiltrating cytotoxic T cells kill cancer cells, and their rate of killing depends on the ratio of cytotoxic T cells and cancer cells. This process also results in the enhanced release of tumor-associated antigens. However, the model assumes that tumor-infiltrating cytotoxic T cells and helper T cells become exhausted by the interaction of PD-1 with ligands on cancer cells and the action of Tregs.

The model also includes the secretion of CCL2 by cancer cells, which is assumed to mediate the recruitment of myeloid-derived suppressor cells (MDSCs) into the tumor compartment. MDSCs are assumed to release arginase-I (Arg-I) and nitric oxide (NO), which inhibit the cytotoxic activity of T cells. TGF-β and Arg-I facilitate the trans-differentiation of helper T cells to Tregs in the tumor.

Finally, the model incorporates the pharmacokinetics and pharmacodynamics of two antibodies: the anti-PD-L1 antibody atezolizumab and the T cell engager antibody cibisatamab. Both antibodies are directly administered into the central compartment. The pharmacokinetics of the antibodies incorporates their clearance from the central compartment, their transport between the central and peripheral/tumor compartments, and their transport from the tumor to the tumor-draining lymph node compartment. The pharmacodynamics of the anti-PD-L1 antibody is modeled by its binding to the PD-L1 on cancer cells and regulatory T cells, which blocks the interactions of PD-1 with PD-L1. The subsequent reduction in the amount of ligand-bound PD-1 decreases the inhibitory action of PD-1 on T cell-mediated killing of cancer cells, which is also modeled as a Hill function. The bispecific T cell engager can bind to CD3 on T cells and CEA on cancer cells, leading to the formation of CEA_TCE_CD3 molecule and enhanced cancer killing by Teff cells. The details on TCE binding module and the equations describing pharmacokinetic of both cibisatamab and atezolizumab are elaborated in the Supplementary Material.

**FIGURE 2:**
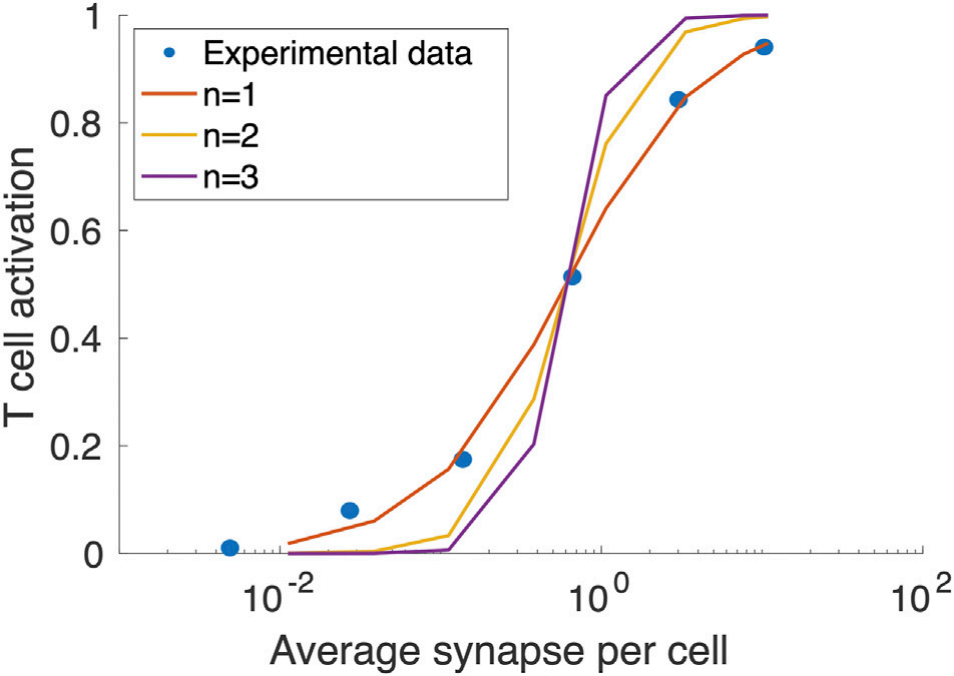
Fitting the Hill function coefficient (n) of T cell activation to concentration on synapse formed per T cells. The blue circles are extracted from (Vyver et al., 2021).

In this study, dynamics of T cells, APCs, tumor-specific neoantigens and tumor-associated self-antigens, immune checkpoints, MDSCs are adapted from (Wang et al., 2021). The tumor growth dynamics with a logistic growth rate and TCE model with binding modifications are based on (Ma et al., 2020b) with modified hill function coefficient of T cell activation fitted to *in vitro* data of cibisatamab, Figure 2. The modules have a total of 131 ordinary differential equations (ODEs), 27 algebraic equations (i.e., repeated assignment rules), 211 parameters, and were created using MATLAB scripts. The online Supplementary Tables S2–S7 include complete listings of model parameters, reactions, algebraic equations, and cellular and molecular species, as well as details on each module.

### 2.2 Virtual patient generation and virtual clinical trial

To create a virtual patient cohort that resembles the clinical population, a subset of model parameters is varied (Supplementary Table S7) while others remain at the baseline level (Supplementary Table S4). Both the baseline values and ranges of selected parameters are based on experimental and clinical data, where available (see Supplementary Table S4 notes for references). However, the distributions of some parameters are not currently available. For those parameters, we have estimated their ranges such that the 95% confidence interval of simulated ORR rate would correspond to the results of the clinical trial (percentage of PR/CR) per RECIST, for each therapy. The model is first initialized with a small number of cells before performing a virtual clinical trial. Using Latin Hypercube Sampling (LHS), the values of selected parameters are randomly generated based on the calibrated parameter distributions, with each parameter set representing a potential virtual patient. If the tumor is able to reach the desired initial tumor size, which corresponds to the pre-treatment tumor size in actual clinical trials and varies among patients, the simulation will proceed to estimate the response to therapy. To avoid generating implausible patients due to uncertainty in parameter ranges, the following physiological parameters were used to screen VPs: tumor diameter, T cell density in the blood, and Teff to Treg ratio.

### 2.3 Statistical analysis

Latin Hypercube Sampling (LHS) and Partial Rank Correlation Coefficient (PRCC) methods are used to perform global uncertainty and sensitivity analyses (Marino et al., 2008) to investigate the effects of varied parameter values on model observations. The virtual patient population is resampled using bootstrap sampling in order to compare model predictions and clinical data. The 95 percentile confidence intervals and bootstrap median are then computed for comparison between model predictions and clinical results. Statistical analyses are carried out via MATLAB 2020a (MathWorks, Natick, MA).

### 2.4 Drug synergy quantification

Using the MuSyC technique (Meyer et al., 2019), the synergy of combination therapy is evaluated for the median behavior of simulated virtual patients. In summary, two parameters representing synergistic potency, α, and synergistic efficacy, *β*, are quantified for a two-dimensional space representative of response to two targeted drugs. The parameter α measures how the presence of another drug affects the effective dose of one drug. When two drugs have synergistic potency (log(α) > 0), the EC50 value decreases due to the addition of the other drug, corresponding to an increase in potency. The percent increase in the effect of a drug combination over the most effective single drug is referred to as the parameter β. For example, in the case of synergistic efficacy (*β* > 0), the maximum effect (E_max_) of combined drugs is greater than the maximum effect of the individual drugs.

## 3 Results

### 3.1 In silico virtual clinical trial of atezolizumab and cibisatamab

For this study, a virtual cohort of 500 patients was created by LHS method, and those who did not reach the desired initial tumor size or with implausible parameter values were regarded as non-patients and excluded from the virtual trial. Filtered virtual patients (VPs) were used for estimating overall response rate (ORR) of colorectal cancer in mono- and combination therapy using atezolizumab and cibisatamab. It is important to note that the same VPs were used in all the cases. The parameters with no experimentally reported values (Supplementary Table S4) were fitted to the outcome of clinical trials NCT02324257 and NCT02650713, with 60 mg cibisatamab QW as a monotherapy treatment and 60 mg cibisatamab QW plus 1200 mg atezolizumab Q3W for combination therapy (Tabernero et al., 2017). The ORRs were calculated for VPs following RECIST 1.1 (Eisenhauer et al., 2009) after 400 days, as summarized in Table 1. In order to compare the simulation results with the actual clinical trials, we have calculated 95% percentile bootstrap confidence intervals (95% CI) of the ORRs by randomly sampling 31 VPs 10,000 times in cibisatamab monotherapy and 25 VPs 10,000 times in combination therapy. MATLAB’s “bootci” function was used for calculation of 95% CI and, the number of selected VPs was chosen based on number of patients in NCT02324257 (31 patients, cibisatamab monotherapy) and NCT02650713 (25 patients, combination therapy). Since the result of atezolizumab was not reported in these trials, the result of atezolizumab monotherapy from the IMblaze370 (NCT02788279) trial with a 2% (95% CI: 0.3–7.8) response rate was used to calibrate the model and compare with the clinical trial results (Eng et al., 2019). Similar to cibisatamab monotherapy, we have randomly sampled 31 patients for calculation of CI in the case of atezolizumab monotherapy.

**TABLE 1:** Overall Response Rate of 60 mg cibisatamab monotherapy, 1200 mg atezolizumab monotherapy and their combination therapy.

Treatment	Simulated ORR (%)	95% CI	Clinical ORR	References
Cibisatamab	8.2	0%–22.6%	6%	Tabernero et al. (2017)
Atezolizumab	3.4	0%–12.9%	2% (IMblaze370 trial)	Eng et al. (2019)
Combination Therapy	11.1	0%–28%	12%	Tabernero et al. (2017)

**FIGURE 3:**
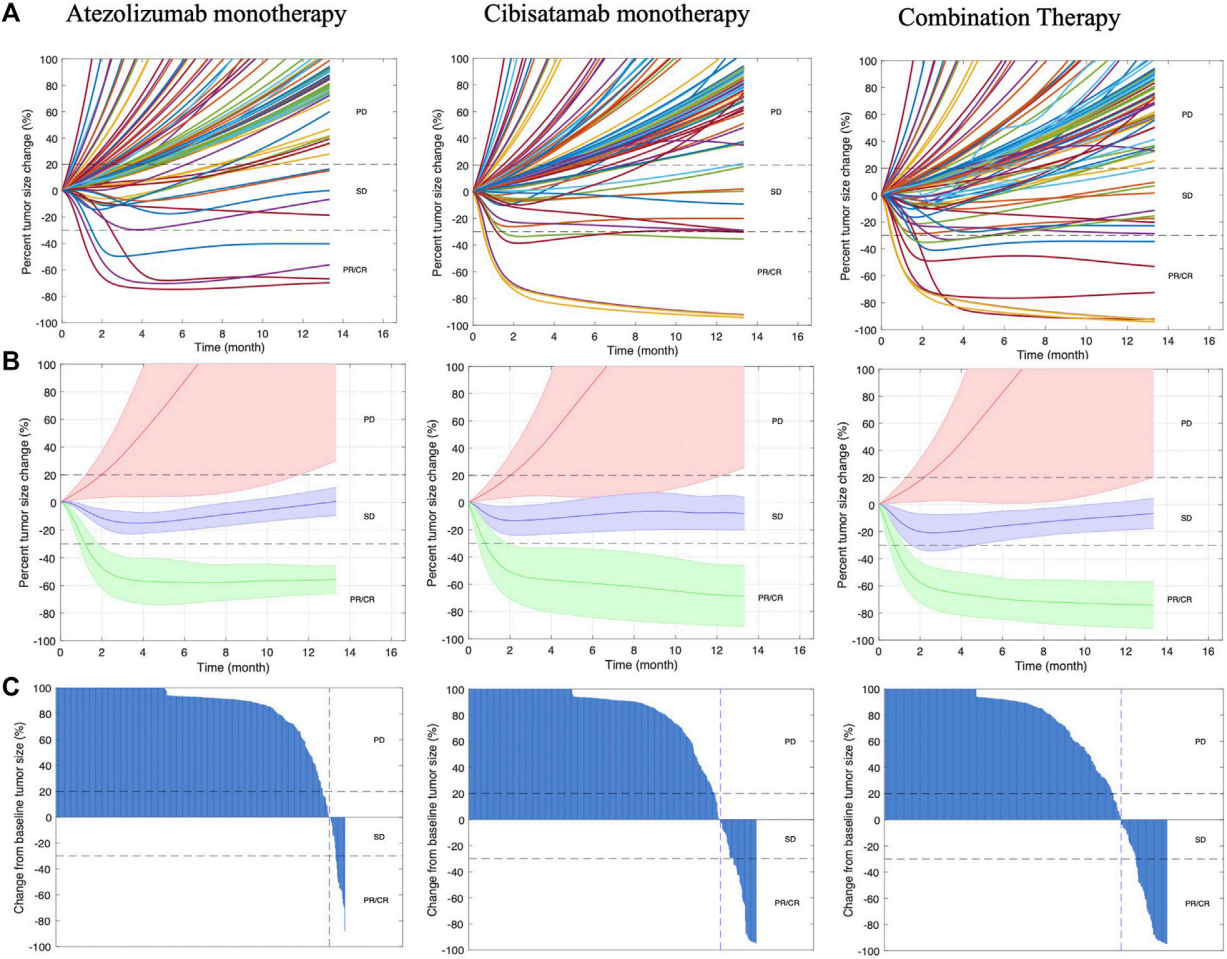
Rate of response in model-predicted tumor diameter of **(A)** 100 randomly selected virtual patients; **(B)** all VPs. Solid line represents the median and shaded area stands for the median absolute error (mad); **(C)** best overall response represented by waterfall plots for all VPs. Response is assessed by RECIST 1.1. CR, complete response; PR, partial response; SD, stable disease; PD, progressive disease.

In order to visualize the dynamics of individual virtual patients and compare different treatments, we plotted the rate of response for all treatment cases as spider plots, showing both the individual variabilities of patients by randomly selecting 100 VPs in Figure 3A, and the median behavior of the virtual population in Figure 3B. In addition, the best overall response is demonstrated by waterfall plots, as shown in Figure 3C.

**FIGURE 4:**
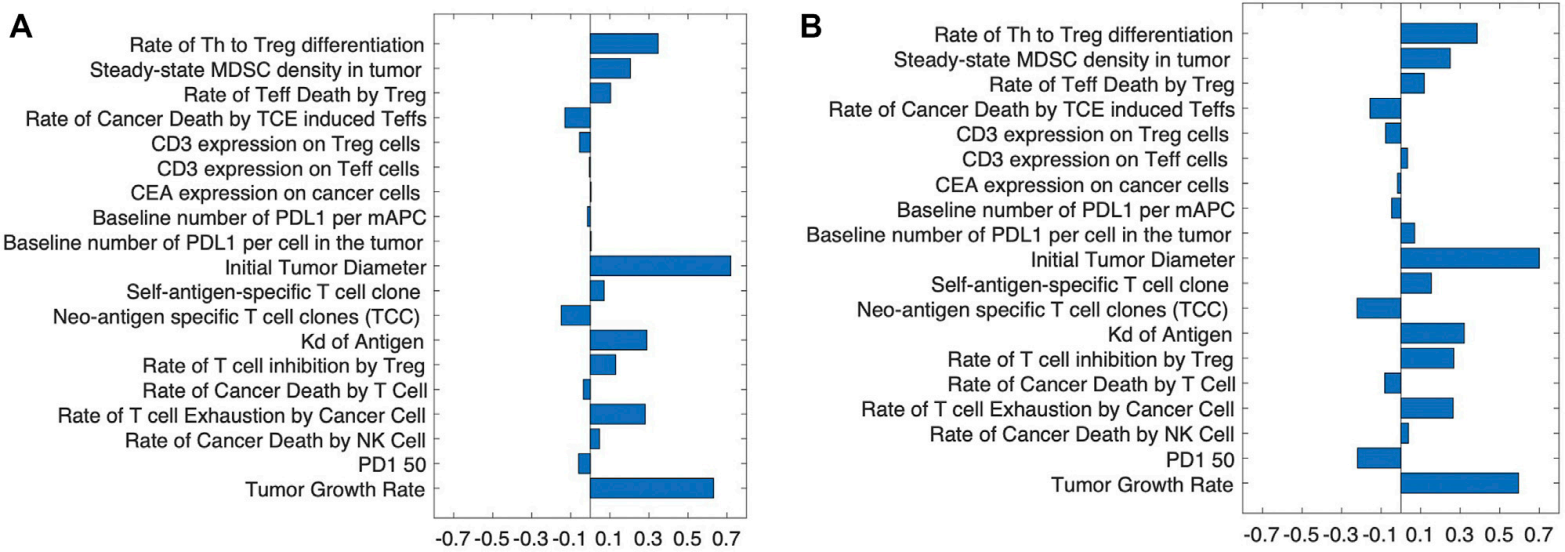
The partial rank correlation coefficient, PRCC, between input parameters and tumor volume after treatment with **(A)** cibisatamab monotherapy and **(B)** combination therapy.

**FIGURE 5:**
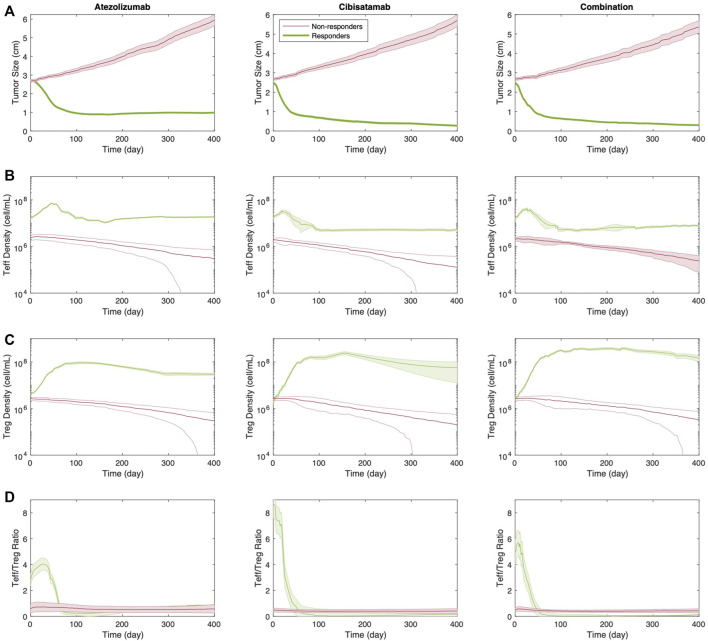
Time profile of **(A)** tumor size; **(B)** Teff cell density; **(C)** Treg cell density; and **(D)** Teff to Treg cells ratio for responders vs. non-responders in monotherapies and combination therapy. Thick line represents the median and shaded area stands for the standard error.

To determine the strength of the correlation between parameters and tumor volume, global uncertainty and sensitivity analysis was performed using PRCC (Figure 4). Tumor volume was significantly positively associated with initial tumor diameter and tumor growth rate in both cibisatamab monotherapy and combination therapy. Moreover, in both cases, neo-antigen specific T cell clones (TCC) were highly negatively associated with tumor volume. To further explore the results, we plot the time profile of tumor size and T cell densities in the tumor compartment in Figure 5. As shown in this figure, the median tumor size is significantly lower in responders, while Teff cell density, Treg cell density and their ratio are higher in responders as expected. Moreover, initial values of Teff cell density is strongly correlated with responder/non-responder status, suggesting that pre-treatment values of Teff density is a predictive biomarker in monotherapies and combination therapy.

### 3.2 Optimization of dose regimen of combination therapy with atezolizumab and cibisatamab by sequential therapy simulations

**FIGURE 6:**
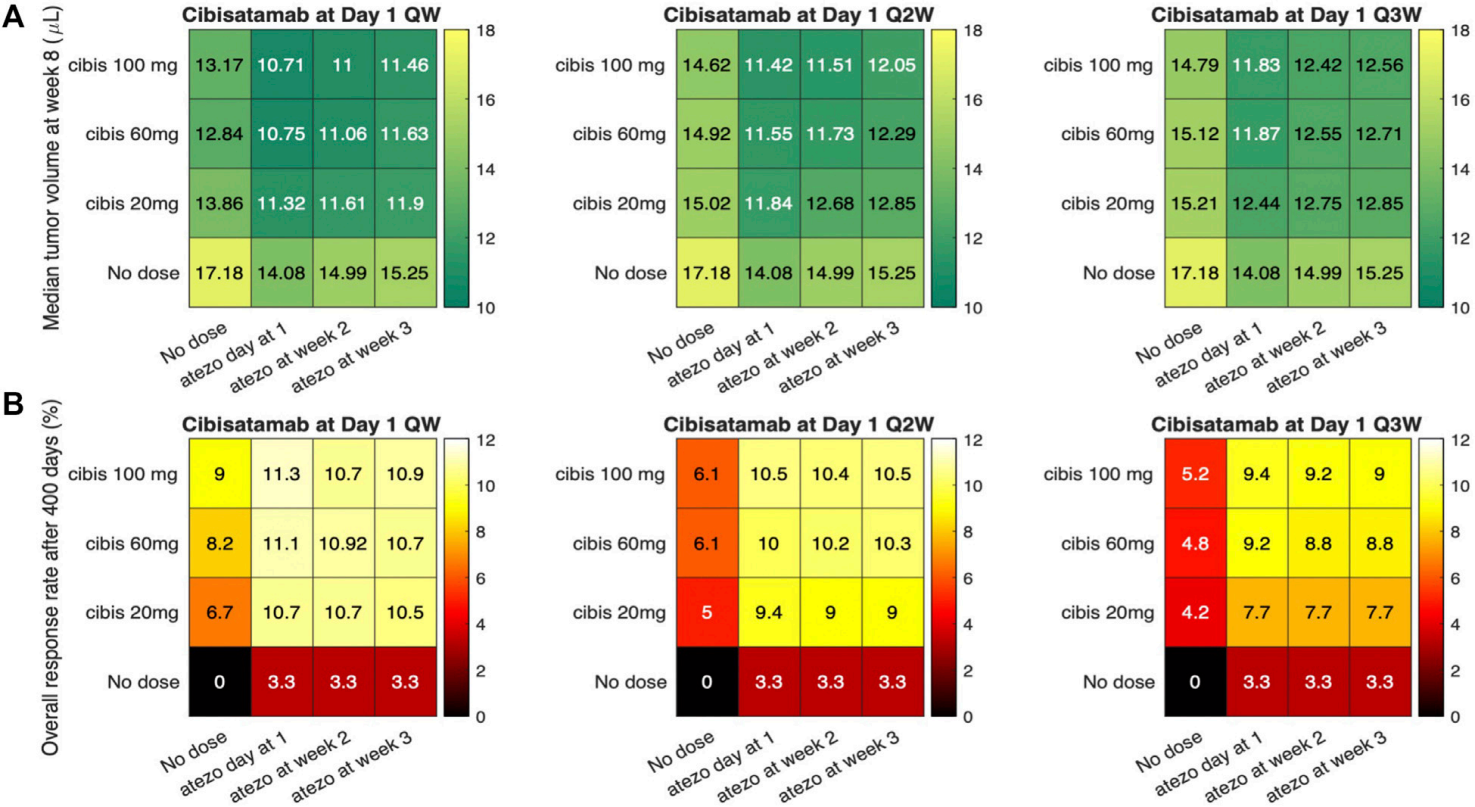
Simulations of sequential therapies using various atezolizumab and cibisatamab dose and schedule. **(A)** represents the median tumor volume after 8 weeks; and **(B)** Overall response rate for each dose regimen.

At the next step, we aim to investigate the possibility of dosage optimization using the current QSP platform, which has been validated by its efficacy prediction of the combination therapy with atezolizumab and cibisatamab. To this end, we conducted 40 different virtual clinical trials, using the same VPs from above, with various cibisatamab and atezolizumab doses and schedules. We have kept the dose and frequency of atezolizumab the same for all the cases, 1,200 mg Q3W, since this is an established dose in clinical trials of colorectal cancer (Tapia Rico et al., 2018). Atezolizumab administration was simulated starting on day 1, week 2 or week 3 after reaching initial tumor diameter, in combination with cibisatamab. Cibisatamab dose size and schedule were selected in the range of 0–100 mg and QW-Q3W, respectively. These selected dose sizes and schedules are in agreement with the ranges used in clinical trials. The median tumor volume at week 8 (the time of first follow up in clinical trial after treatment) and ORR for each combination at the end of treatment are reported in Figure 6. We aimed to determine whether treatment outcomes differed between early in treatment results and end-point results. To achieve this, we primarily measured the median tumor volume, as the overall response rate (ORR) may not fully reflect individual patient dynamics until the end of the treatment.

Overall, by considering both ORR and median tumor volume, the result shows that concurrent combination therapy has slightly better response compared to sequential therapies. Although the highest efficacy is observed for weekly administration of cibisatamab, which is the same frequency used in clinical trials, the result of simulation shows that biweekly (Q2W) and triweekly (Q3W) administration of cibisatamab can have similar efficacy, which may be beneficial to reduce the toxicity associated with bispecific antibodies.

### 3.3 Quantification of drug synergy

**FIGURE 7:**
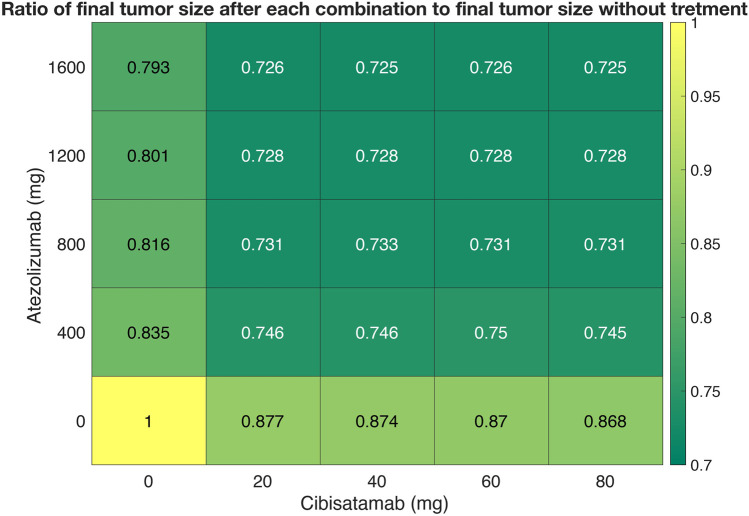
A dose-response heatmap for atezolizumab within the range of 0–1600 mg and cibisatamab in the range of 0–80 mg.

Next, we used the QSP model to quantify the synergy of combination therapy with atezolizumab and cibisatamab. 25 different simulations were conducted for various combinations of drugs concentration, with cibisatamab in the range of 0–80 mg and atezolizumab within the range of 0–1600 mg for the same VPs in each simulation. For each combination, the ratio of final tumor size to tumor size at the conditions with no drug at the end of 400 days, was calculated as the metric of response. Then, a two-dimensional heatmap was plotted for the results, Figure 7. Quantification of the synergy using multidimensional synergy of combinations (MuSyC) technique suggests a small synergistic efficacy *β*_obs_ = 0.072, as well as a small synergistic potency log (α_2_) = 0.064.

## 4 Discussion

TCEs have become an important part of the therapeutic research strategy to treat cancer (Dahlén et al., 2018; Zhou et al., 2021). They enable a powerful mode of action by activating T cells through the creation of artificial immune synapses (Morcos et al., 2020). Explorative preclinical and emerging clinical data indicate a potential for enhanced efficacy and reduced systemic toxicity. However, TCEs are a complex modality with challenges to overcome in early clinical trials, including the selection of relevant starting doses. “Dosing strategy plays a crucial role in determining the therapeutic window of TCEs because of the desire to maximize therapeutic efficacy in the context of known mechanism-related adverse events, such as cytokine release syndrome (CRS) and neurological adverse events” (Betts and van der Graaf, 2020). Moreover, other drug combinations with T cell engagers have been a promising approach to treat cancers. While comprehensive drug combination tests are effective for identifying novel synergistic drug combinations, measuring all possible combinations is challenging due to the size of potential therapeutic agents and cell lines. Mechanistic modeling approaches like quantitative system pharmacology (QSP) models are powerful tools that can be used to integrate diverse data to predict/refine clinical dosing regimens and design trials to optimize efficacy (Jafarnejad et al., 2019; Hosseini et al., 2020; Sové et al., 2020; Sové et al., 2022; Wang et al., 2022). Modeling can be used to guide rational decision making, to inform precision medicine strategies, and to increase overall efficiency of the oncology clinical development process (Betts and van der Graaf, 2020; Gibbs et al., 2020).

In this study, we extend our previously developed QSP platform (Ma et al., 2020a) to study the combination therapy of an immune checkpoint inhibitor, atezolizumab, and a T cell engager, cibisatamab. Following recent developments in kinetic modeling of bispecific antibodies (Vauquelin and Charlton, 2013; Schropp et al., 2019), we simplified the binding of cibisatamab to CEA, by considering one binding arm with a newly added parameter to account for the avidity of cibisatamab and thus modifying the binding affinity between the second target and cibisatamab bound to the first target. We calibrated the model by fitting the model to the experimental data of level of T cell activation as a function of average synapse per cell from (Vyver et al., 2021). We have also adopted the dynamics of T cells, helper T cells, APCs, tumor-specific neoantigens and tumor-associated self-antigens, immune checkpoints and MDSCs from our study of TNBC (Wang et al., 2021).

Using the model calibrated against clinical data, we performed a series of *in silico* clinical trials to investigate the optimal dose schedule of atezolizumab and cibisatamab for colorectal cancer. The results suggest that concurrent combinations result in higher ORRs (and smaller tumor size) than sequential combinations. Although the highest efficacy is observed for weekly administration of cibisatamab, which is the same frequency used in clinical trial, the results of simulations show that biweekly (Q2W) and triweekly (Q3W) administration of cibisatamab can have similar efficacy to weekly with potentially less toxicity and adverse events associate with CRS in T cell engagers (Yu and Wang, 2019).

To investigate how the presence of one drug would affect the efficacy and potency of the other drug in combination therapy, we investigated the drug synergy quantification using MuSyC method developed by (Meyer et al., 2019). The results of our simulations showed insignificant synergy of potency and efficacy for combination therapy of atezolizumab and cibisatamab. This could be due to the fact that these two drugs have independent mechanisms of action. In this study, we have used the ratio of tumor size at the end of simulation following treatment to tumor size at the end of the simulation with no treatment as the metric of response. However, other metrics like ORR, duration of response or T cell densities might be other potential metrics to further investigate the drug synergy in combination therapy. Moreover, the drug synergy quantification based on these traditional statistical models may lack high power and accuracy measurement due to small data size. In future, and with availability of additional data the machine learning techniques of drug synergy quantifications may bring many advantages, including high accuracy, ability to model non-linear effects, and robustness to parameter assumptions (Preuer and Lewis, 2018; Liu and Xie, 2021; Zhang et al., 2021; Tang and Gottlieb, 2022).

One of the major challenges in QSP models is the parameter estimations due to high complexity of the models. Generation of virtual patients with the goal of establishing a reliable and effective algorithm is an ongoing research in the field of pharmacology modeling (Allen et al., 2016; Rieger et al., 2018). In this study, most of the parameters are estimated using experimental data for colorectal cancer and validated by comparing the results to response rate of patients in clinical trial. However, the importance of some parameters like the rate of Teff suppression by activated Tregs due to bispecific T cell engagers and their influence on the result of combination therapy remains to be explored.

Here, we used QSP modeling to perform *in silico* clinical trials of atezolizumab and cibisatamab to study optimization of dose and schedule in combination therapy and drug synergy quantification. This model can be extended to study other bispecific T cell engagers and immune checkpoint inhibitors in colorectal and other cancers if sufficient data for parameter recalibration and model validation are available. Also, the QSP approach can be used in model-informed drug design (MIDD) and design of clinical trials and provide regulatory assistance (Azer et al., 2021; Bai et al., 2021).

## Data Availability

The datasets presented in this study can be found in online repositories. The names of the repository/repositories and accession number(s) can be found in the article/Supplementary Material. The model code and MATLAB script used in this study are available at http://dx.doi.org/10.17632/crk9tmnmb7.1.
