# Supplementary material for: Using quantitative systems pharmacology modeling to optimize combination therapy of anti-PD-L1 checkpoint inhibitor and T cell engager
Source: Front Pharmacol. 2023 Jun 20;14:1163432. doi: 10.3389/fphar.2023.1163432 (PMC10318535; doi:10.3389/fphar.2023.1163432)
Supplement: Supplementary file 1 [file DataSheet1.pdf]

Supplementary Material

Using quantitative systems pharmacology modeling to optimize combination therapy of anti-PD-L1 checkpoint inhibitor and T cell engager

Samira Anbari[1*], Hanwen Wang[1], Yu Zhang[1], Jun Wang[2], Minu Pilvankar[2], Masoud Nickaeen[2], Steven Hansel[2], Aleksander S. Popel[1,3]

[1] Department of Biomedical Engineering, Johns Hopkins University School of Medicine, Baltimore, MD, USA

[2]Biotherapeutics Discovery Research, Boehringer Ingelheim Pharmaceuticals Inc, Ridgefield, Connecticut, USA

[3] Department of Oncology, and the Sidney Kimmel Cancer Center, Johns Hopkins University School of Medicine, Baltimore, MD, USA

*** Correspondence:** Samira Anbari :smeybod1@jhmi.edu

Supplementary Information

1- TCE binding module

The dynamics of TCE binding to cancer calls and T cells are described in this section following equations 1-3. In summary, TCE can bind either to CEA on cancer cells or CD3 on T cells (Teff or Treg) cells to form the dimers CEA_TCE or CD3_TCE respectively (Eqs. 1, 2-1, 2-2). For simplicity, we assume that cibisatamab has only one binding arm for CEA. After the dimers are formed, they can subsequently bind to CD3 and CEA to form the final molecule, CEA_TCE_CD3 (Eqs. 3-1, 3-2). A summary of all parameters used in these equations with their units and description are provided in Table S1.

Table S1 : Summary of the parameters and variables used in the TCE binding module

Parameter/Variable	Unit	Description
TCE	M	TCE (cibisatamab) concentration in the tumor compartment
C_CEA_total	molecule	Total number of CEA per cancer cell
Teff_CD3_total	molecule	Total number of CD3 per Teff

Treg_CD3_total	molecule	Total number of CD3 per Treg
SA_Ccell	μm^2	Surface area of a cancer cell
SA_Tcell	μm^2	Surface area of a T cell
D_{CEA}	$\text{molecule} * \mu m^{-2}$	CEA density on cancer cells calculated as $D_{CEA} = \frac{\text{C\_CEA\_total}}{SA\_Ccell}$
$D_{TeffCD3}$	$\text{molecule} * \mu m^{-2}$	CD3 density on Teff cells calculated as $D_{TeffCD3} = \frac{\text{Teff\_CD3\_total}}{SA\_Tcell}$
$D_{TregCD3}$	$\text{molecule} * \mu m^{-2}$	CD3 density on Treg cells calculated as $D_{TregCD3} = \frac{\text{Treg\_CD3\_total}}{SA\_Tcell}$
D_{syn}	nm	Immunological synapse gap
N	$\text{molecule} * \text{mole}^{-1}$	Avogadro constant
k_{on,CEA_TCE}	$M^{-1} * s^{-1}$	Association constant of TCE and CEA binding
k_{off,CEA_TCE}	s^{-1}	Dissociation constant of TCE and CEA binding
$k_{on,CD3\_TCE}$	$M^{-1} * s^{-1}$	Association constant of TCE and CD3 binding
$k_{off,CD3\_TCE}$	s^{-1}	Dissociation constant of TCE and CD3 binding

Following other models of bispecific antibodies (Vauquelin and Charlton 2013; Schropp et al. 2019), a parameter called *f* is added to count for avidity of TCE to two targets, which implies that binding to the first target affects the binding affinity to the second target (Eqs. 3-1, 3-2). The formation of CEA_TCE_CD3 in the immunological synapse will cause enhanced cancer killing by Teff since more Teff cells are triggered by TCE in an MHC-independent manner. Treg will also be activated by TCEs to exhibit an immunoregulatory function as was suggested by experimental data (Koristka et al. 2015, 2012), which is achieved by suppressing the activity of Teff cells in this model.

$$\frac{d(CEA\_TCE)}{dt} = k_{on,CEA\_TCE} \cdot D_{CEA} \cdot TCE - k_{off,CEA_{TCE}} \cdot D_{CEA_{TCE}} - \frac{k_{on,CD3_{TCE}}}{D_{syn} * N} \cdot D_{CEA_{TCE}} \cdot D_{TeffCD3} + k_{off,CD3_{TCE}} \cdot D_{CEA_{TCE_{TeffCD3}}} - \frac{k_{on,CD3_{TCE}}}{D_{syn} * N} \cdot D_{CEA_{TCE}} \cdot D_{TregCD3} + k_{off,CD3_{TCE}} \cdot D_{CEA_{TCE_{TregCD3}}} \quad (1)$$

$$\frac{d(TeffCD3\_TCE)}{dt} = k_{on,CD3\_TCE} \cdot D_{TeffCD3} \cdot TCE - k_{off,CD3\_TCE} \cdot D_{TeffCD3\_TCE} - \frac{k_{on,CEA\_TCE}}{D_{syn} * N} \cdot D_{TeffCD3\_TCE} \cdot D_{CEA} + k_{off,CEA\_TCE} \cdot D_{CEA\_TCE\_TeffCD3} \quad (2\text{-}1)$$

$$\frac{d(TregCD3\_TCE)}{dt} = k_{on,CD3\_TCE} \cdot D_{TregCD3} \cdot TCE - k_{off,CD3\_TCE} \cdot D_{TregCD3\_TCE} - k_{on,CEA\_TCE} \cdot D_{TregCD3\_TCE} \cdot D_{CEA} + k_{off,CEA\_TCE} \cdot D_{CEA\_TCE\_TregCD3} \quad (2\text{-}2)$$

$$\frac{d(CEA\_TCE\_TeffCD3)}{dt} = \frac{k_{on,CEA\_TCE}}{f * D_{syn} * N} \cdot D_{TeffCD3\_TCE} \cdot D_{CEA} - k_{off,CEA\_TCE} \cdot D_{CEA\_TCE\_TeffCD3} + \frac{k_{on,CD3\_TCE}}{f} \cdot D_{CEA\_TCE} \cdot D_{TeffCD3} - k_{off,CD3\_TCE} \cdot D_{CEA\_TCE\_TeffCD3} \quad (3\text{-}1)$$

$$\frac{d(CEA\_TCE\_TregCD3)}{dt} = \frac{k_{on,CEA\_TCE}}{f * D_{syn} * N} \cdot D_{TregCD3\_TCE} \cdot D_{CEA} - k_{off,CEA\_TCE} \cdot D_{CEA\_TCE\_TregCD3} + \frac{k_{on,CD3\_TCE}}{f} \cdot D_{CEA\_TCE} \cdot D_{TregCD3} - k_{off,CD3\_TCE} \cdot D_{CEA\_TCE\_TregCD3} \quad (3\text{-}2)$$

The number of bound CEA_TCE_CD3 was translated to cancer cell killing rate by activation of Teff cells using a Hill equation. The Hill function coefficient was calculated by fitting the T cell activation as a function of average number of CEA_TCE_TeffCD3 per Teff cell to experimental data of MKN45 published in the study by Van De Vyver et. al. (van de Vyver et al. 2021) (Figure 2). The equations of cancer killing by TCE induced Teffs are provided by Ma et. al. (Ma, Wang, Sové, et al. 2020)

2- Pharmacokinetics

Pharmacokinetic of both cibisatamab and atezolizumab were modelled following the same physiologically-based pharmacokinetic model as described by (Jafarnejad et al. 2019). The equations describing drug PK has presented below, equations 4-7. A_i indicate antibody (either cibisatamab or atezolizumab) concentration. V_i is the compartment volume, Q_i is the volumetric flow rate between the central and the corresponding compartment, q_{LD} is the rate of lymphatic drainage from tumor to TDLNs and from TDLNs to central, and CL is clearance rate. Subscripts C, P, LN, and T represent the central, peripheral, tumor-draining lymph node, and tumor compartments, respectively.

The PK parameters of cibisatamab and atezolizumab in this model has been previously estimated by Ma et. al. 2020 fitted to standard pharmacokinetic two-compartment model. Cibisatamab PK parameters were fitted to reported plasma concentration at dose levels of 80, 160, 200, 300, 400 mg. Atezolizumab PK parameters were fitted to reported plasma concentration at dose levels of 1, 3, 10, 15 mg/kg and 1200mg. The parameters are provided in the Table S4. The simulated concentrations of atezolizumab (1200mg Q3W) and cibisatamab (60mg QW) in each compartment are presented in Fig. S1.

$$V_C \frac{dA_C}{dt} = Q_P(A_P - A_C) + Q_{LN}(A_{LN} - A_C) + Q_T(A_T - A_C) + q_{LD} * V_T * A_{LN} - CL * A_C \quad (4)$$

$$V_P \frac{dA_P}{dt} = Q_P(A_C - A_P) \quad (5)$$

$$V_T \frac{dA_T}{dt} = Q_T(A_C - A_T) - q_{LD} * V_T * A_T \quad (6)$$

$$V_{LN} \frac{dA_{LN}}{dt} = Q_{LN}(A_C - A_{LN}) + q_{LD} * V_T * A_T - q_{LD} * V_T * A_{LN} \quad (7)$$

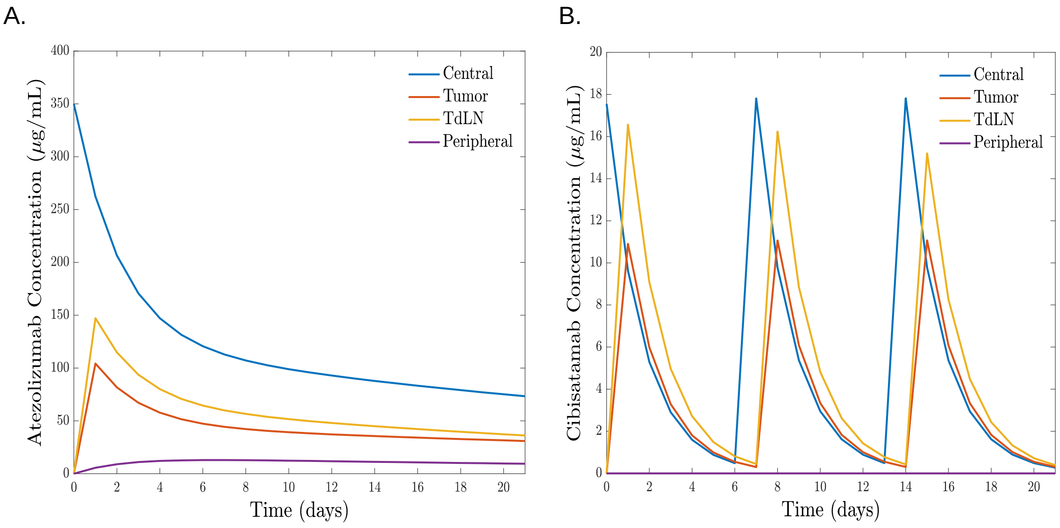

Figure S1. The concentration of A) atezolizumab (1200mg Q3W) and B) cibisatamab (60mg QW) in central, Tumor, Tumor Draining Lymph Node (TdLN), and Peripheral compartments.

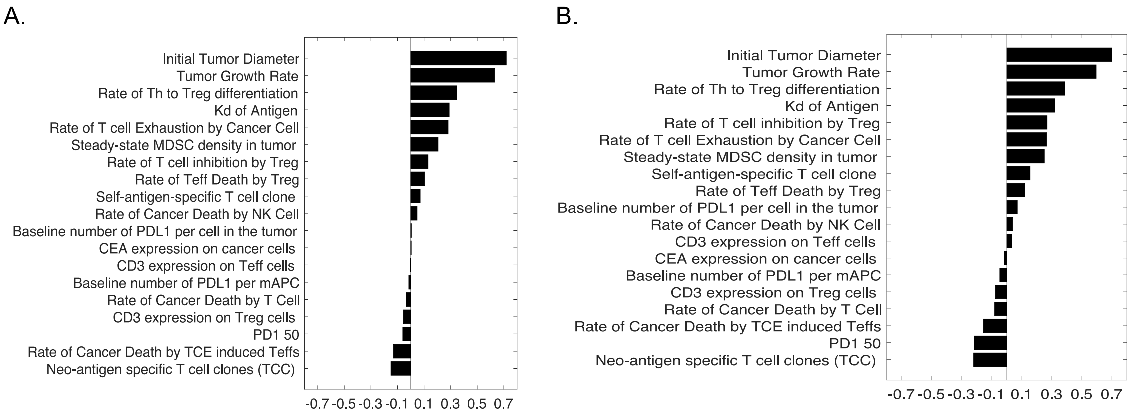

Figure S1. The partial rank correlation coefficient, PRCC, between input parameters and tumor volume after treatment with (A) cibisatamab monotherapy and (B) combination therapy.

References

Jafarnejad, Mohammad, Chang Gong, Edward Gabrielson, Imke H. Bartelink, Paolo Vicini, Bing Wang, Rajesh Narwal, Lorin Roskos, and Aleksander S. Popel. 2019. “A Computational Model of Neoadjuvant PD-1 Inhibition in Non-Small Cell Lung Cancer.” *AAPS Journal* 21 (5). https://doi.org/10.1208/s12248-019-0350-x.

Koristka, Stefanie, Marc Cartellieri, Claudia Arndt, Anja Feldmann, Barbara Seliger, Gerhard Ehninger, and Michael P. Bachmann. 2015. “Tregs Activated by Bispecific Antibodies: Killers or Suppressors?” *OncoImmunology* 4 (3): 1–3. https://doi.org/10.4161/2162402X.2014.994441.

Koristka, Stefanie, Marc Cartellieri, Anke Theil, Anja Feldmann, Claudia Arndt, Slava Stamova, Irene Michalk, et al. 2012. “Retargeting of Human Regulatory T Cells by Single-Chain Bispecific Antibodies.” *The Journal of Immunology* 188 (3): 1551–58. https://doi.org/10.4049/jimmunol.1101760.

Ma, Huilin, Hanwen Wang, Richard J. Sove, Mohammad Jafarnejad, Chia Hung Tsai, Jun Wang, Craig Giragossian, and Aleksander S. Popel. 2020. “A Quantitative Systems Pharmacology Model of T Cell Engager Applied to Solid Tumor.” *AAPS Journal* 22 (4): 1–16. https://doi.org/10.1208/s12248-020-00450-3.

Ma, Huilin, Hanwen Wang, Richard J. Sové, Jun Wang, Craig Giragossian, and Aleksander S. Popel. 2020. “Combination Therapy with T Cell Engager and PD-L1 Blockade Enhances the Antitumor Potency of T Cells as Predicted by a QSP Model.” *Journal for ImmunoTherapy of Cancer* 8 (2): 1–11. https://doi.org/10.1136/jitc-2020-001141.

Schropp, Johannes, Antari Khot, Dhaval K. Shah, and Gilbert Koch. 2019. “Target-Mediated Drug Disposition Model for Bispecific Antibodies: Properties, Approximation, and Optimal Dosing Strategy.” *CPT: Pharmacometrics and Systems Pharmacology* 8 (3): 177–87. https://doi.org/10.1002/psp4.12369.

Vauquelin, Georges, and Steven J. Charlton. 2013. “Exploring Avidity: Understanding the Potential Gains in Functional Affinity and Target Residence Time of Bivalent and Heterobivalent Ligands.” *British Journal of Pharmacology* 168 (8): 1771–85. https://doi.org/10.1111/bph.12106.

Vyver, Arthur J. van de, Tina Weinzierl, Miro J. Eigenmann, Nicolas Frances, Sylvia Herter, Regula B. Buser, Jitka Somandin, et al. 2021. “Predicting Tumor Killing and T-Cell Activation by t-Cell Bispecific Antibodies as a Function of Target Expression: Combining in Vitro Experiments with Systems Modeling.” *Molecular Cancer Therapeutics* 20 (2): 357–66. https://doi.org/10.1158/1535-7163.MCT-20-0269.
